# Dose–Response Meta-Analysis of Corticosteroid Effects in SARS Outbreak: A Model for Risk Stratification and Screening Strategy for Osteonecrosis of Femoral Head Post-Corticosteroid Therapy for COVID-19

**DOI:** 10.3390/life13040907

**Published:** 2023-03-29

**Authors:** Sathish Muthu, Madhan Jeyaraman, Preethi Selvaraj, Naveen Jeyaraman, Anish G. Potty, Ashim Gupta

**Affiliations:** 1 Orthopaedic Research Group, Coimbatore 641045, Tamil Nadu, India; 2 Department of Orthopaedics, Government Medical College and Hospital, Dindigul 624003, Tamil Nadu, India; 3 Department of Orthopaedics, Dr. MGR Educational and Research Institute, ACS Medical College and Hospital, Chennai 600056, Tamil Nadu, India; 4 South Texas Orthopaedic Research Institute (STORI Inc.), Laredo, TX 78045, USA; 5 Department of Community Medicine, Dr. MGR Educational and Research Institute, Faculty of Medicine, Sri Lalithambigai Medical College and Hospital, Chennai 600095, Tamil Nadu, India; 6 Department of Orthopaedics, Shri Sathya Sai Medical College and Research Institute, Sri Balaji Vidyapeeth, Chengalpet 603108, Tamil Nadu, India; 7 Future Biologics, Lawrenceville, GA 30043, USA; 8 Regenerative Orthopaedics, Noida 201301, Uttar Pradesh, India

**Keywords:** COVID, corticosteroids, osteonecrosis of femoral head, meta-analysis, dose–response meta-analysis, screening, SARS

## Abstract

Corticosteroids (CS) have been used in the management regimens for COVID-19 disease to mitigate the cytokine storm and ill effects of the pulmonary inflammatory cascade. With the rampant use of CS, clinicians started reporting the occurrence of osteonecrosis of the femoral head (OFH). In this systematic review, we aim to analyze the literature and identify the definitive cumulative dose and duration of CS needed for the development of OFH based on the SARS model and generate a risk-based screening recommendation for OFH in convalescent COVID-19 patients to facilitate early identification and management. An electronic database search was conducted until December 2022 in PubMed, Web of Science, Embase, and CNKI (China Knowledge Resource Integrated Database). Studies involving CS therapy and osteonecrosis data in SARS patients were included. Three authors independently extracted the data from the included studies and a dose–response meta-analysis was performed for various doses and duration of CS utilized in the included studies. We selected 12 articles with 1728 patients in the analysis. The mean age was 33.41 (±4.93) years. The mean dosage of CS administered was 4.64 (±4.7) g which was administered for a mean duration of 29.91 (±12.3) days. The risk of osteonecrosis increases at pooled OR of 1.16 (95% CI 1.09–1.23, *p* < 0.001) per 2.0 g increase in the cumulative dose of CS usage. Similarly, the risk increases at pooled OR of 1.02 (95% CI 1.01–1.03, *p* < 0.001) per 5 days of increase in the cumulative duration of CS usage. A cumulative dosage of 4 g and a duration of 15 days were determined as the critical cut-off for the non-linear dose–response relationship observed. Appropriate and frequent screening of these individuals at regular intervals would help in the identification of the disease at an early stage in order to treat them appropriately.

## 1. Introduction

The outbreak of a “pneumonia of unknown etiology” in the province of Wuhan in China notified on 31 December 2019, was due to SARS-CoV-2. Due to the rampant spread across 195 countries, the World Health Organization (WHO) declared Coronavirus disease 2019 (COVID-19) as a pandemic on 11 March 2020. The COVID-19 pandemic created not only a global medical challenge in diagnosing and treating the disease as the SARS-CoV-2 genome mutates frequently but also a greater impact on the world’s socioeconomic burden. After the second wave of the COVID-19 disease, various researchers reported varied presentations of post-COVID-19 sequelae, which posed a greater challenge among healthcare systems around the world.

Corticosteroids (CS) have been used in the management regimens for COVID-19 disease to mitigate the cytokine storm and ill effects of the pulmonary inflammatory cascade. With the rampant use of CS, clinicians started reporting the occurrence of osteonecrosis of the femoral head (OFH) [1]. The mechanism of OFH has been ascribed to raised intra-osseous pressure and decreased bone perfusion which might be resulting from either the disease-induced hypercoagulability or the CS-induced increase in the lip globules in the bone marrow. CS-induced osteocyte apoptosis also disrupts the osteocyte-lacunar-canalicular system and leads to joint collapse [2]. OFH and its resultant secondary arthritis require long-term medical and repeated surgical procedures to salvage the native joint and preserve its function before a replacement procedure can be planned [3].

During the Severe Acute Respiratory Syndrome (SARS) epidemic, CS proved to reduce pulmonary inflammation and reduce the mortality of SARS cases [4]. A total of 18 (23.1%) out of 78 cases developed OFH in the post-SARS phase in the Chinese population [5]. An increased rate of OFH was observed when CS were administered at >20 mg/day. Every 10 mg/day increase in the dose of CS was associated with a 3.6% rise in the development of OFH [6]. Various studies have shown that the occurrence of OFH as the sequelae of SARS was mainly due to the iatrogenic rampant usage of CS in their management. On similar grounds, reports of bilateral OFH post-COVID-19 are on the rise [1,3,7,8,9]. Aggarwala et al., in their series, noted OFH at a mean duration of 59 days (range: 45 to 67 days) at a mean dose of 759 mg prednisolone (range: 400 to 1250 mg) [10]. Similarly, a wide variability is noted in the reported duration and cumulative dose of CS from as low as 45 days to 1 year and 700 mg to 12 g, respectively, necessary for the development of OFH as a sequalae to COVID-19 [11,12,13]. There exists a gap in the existing literature on the definitive duration and the cumulative dose of the CS that is necessary for the development of OFH from COVID-19. Hence, we tried to exploit the data on the incidence of OFH from the SARS pandemic with their individual CS utilization parameters such as dose and duration to develop a screening strategy for the post-COVID-19 era. Having known the duration and dose of CS used, we could categorize the COVID-19-affected individuals based on their cumulative CS utilized in their COVID-19 management to appropriate screening protocols to identify the OFH early and embark on appropriate treatment measures as early as possible [14].

In this systematic review, we aim to analyze the literature and identify the definitive dose and duration of CS needed for the development of OFH based on the SARS model and generate a risk-based screening recommendation for OFH in convalescent COVID-19 patients to facilitate their early identification and management.

## 2. Materials and Methods

### 2.1. Search Criteria

The systematic review was conducted according to the recommendations of PRISMA and the reporting criteria for the guidelines for systematic review as presented in Table S1. The methods for statistical analysis and inclusion criteria were specified in a protocol and documented.

**Figure 1:**
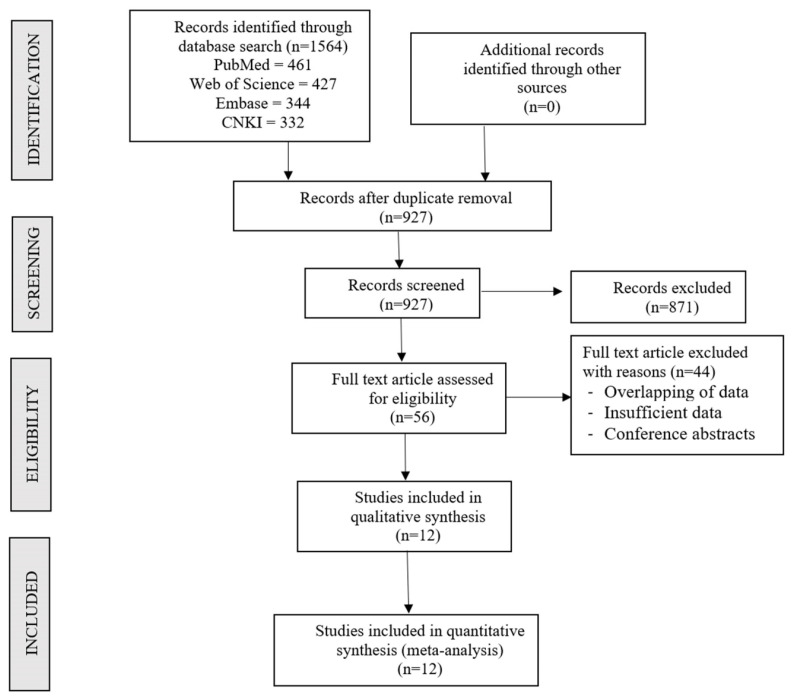
PRISMA flow diagram of the included studies.

An electronic search was conducted until December 2022 including articles from January 2003 to December 2022 using databases such as PubMed, Web of Science, Embase, and CNKI (China Knowledge Resource Integrated Database). Studies involving CS therapy and osteonecrosis data in SARS patients were included. The terms used for the search included: “severe acute respiratory syndrome”, “SARS”, “glucocorticoid(s)”, “corticosteroid(s)”, “steroid”, “cortisol(s)”, “prednisolone(s)”, and “methylprednisolone(s).” The sample search strategy of one of the included databases is presented in Supplementary Materials. We also examined and carried out a forward search on the reference lists of the articles. A detailed study selection flow diagram is given in Figure 1. The studies that were included had to meet the following PICOS criteria:

Population: Patients recovered from SARS;
Intervention: CS therapy with complete therapy regimens provided;
Comparator: Control group without CS therapy;
Outcome: OFH;
Study Design: Cohort or case-control.

The studies that have not addressed SARS patients who have not received CS therapy or without complete CS regimes were excluded. A definitive diagnosis of OFH should be based on Magnetic Resonance Imaging (MRI) in the included studies. OFH has been defined in MRI as subchondral or intra-medullary area necrosis, with a distinct marginal rim of low signal intensity covering the medullary fat of T1-weighted images. Studies were excluded if mixed interventions were used in the treatment groups. Two authors performed an independent and exclusive selection of studies and discrepancies in study selection were resolved by discussion until a consensus was achieved.

### 2.2. Data Extraction

Three reviewers independently retrieved relevant data from the articles to be included in the analysis. The following information was extracted:

Study characteristics: authors, year of publication, country;
Baseline characteristics: mean age, study design, number of patients enrolled;
Efficacy Outcomes: dosage, duration, and follow-up in months;
Safety Outcomes: adverse events.

We attempted to contact the original author first for missing data. If the methodology was unclear, the corresponding author was contacted to determine the type and dosage of glucocorticoid used in their study. Any disagreements in data collection were resolved through discussion until a consensus was reached.

### 2.3. Risk of Bias and Quality Assessment

Three reviewers independently assessed the methodological quality of the included studies using the Cochrane Collaboration’s ROBINS tool for non-randomized studies that have seven domains of assessment.

### 2.4. Statistical Analysis

We used the odds ratio (OR) and 95% confidence intervals (CI) of developing OFH for the individual dose provided in the study. If the study did not report the OR and 95% CI, we calculated the OR and 95% CI based on the reported events of OFH for the individual dose range with the control group as the reference population. Similarly, the OR and 95% CI for the duration of the CS treatment were also utilized for analysis. If the studies reported only the range of CS dose or treatment duration, we used the values of the upper and lower bounds to identify the median dosage for the category. If the highest value was open-ended, we assumed its interval length to be the same as the adjacent interval and when the lowest value was open-ended, we assigned an average value of the upper bound and zero to identify the median dosage for analysis.

We identified the slope and standard error of the regression curve fitting the various dosage of CS therapy utilized in the individual studies under analysis. We performed a single-stage dose–response meta-analysis using the slope and standard errors obtained from the individual studies as described by Crippa et al. [15]. Forest plots were used to describe the effect of the intervention on the outcome analyzed. We used the i^2^ test to study inter-study heterogeneity [16]. We used a fixed effects model when i^2^ value < 50% and *p*-value > 0.1. Otherwise, a random effects model was utilized. We estimated the non-linear polynomial dose–response relationship of the dose and duration of CS treatment using a restricted cubic spline model [17]. A *p*-value < 0.05 was considered significant. We conducted a sensitivity analysis to explore the robustness of the results by exploring the heterogeneity when it existed in the results of the analysis. We used a funnel plot for cumulative dosage and treatment duration to analyze the publication bias. Analysis was performed in Stata (16.1, Stata Corp LLC).

## 3. Results

A total of 1564 articles were retrieved from the literature search, which upon duplicate removal resulted in 927 articles. After the initial title and abstract screening process, 871 of them were excluded resulting in 56 potential articles for full-text screening. Forty-four articles were removed due to insufficient data, overlapping data, and studies published only as conference abstracts. Finally, we selected 12 articles [18,19,20,21,22,23,24,25,26,27,28,29] with 1728 patients for inclusion in the analysis. The PRISMA flow diagram of the selection process was presented in Figure 1. The general characteristics of the included studies are summarized in Table 1.

**Figure 2:**
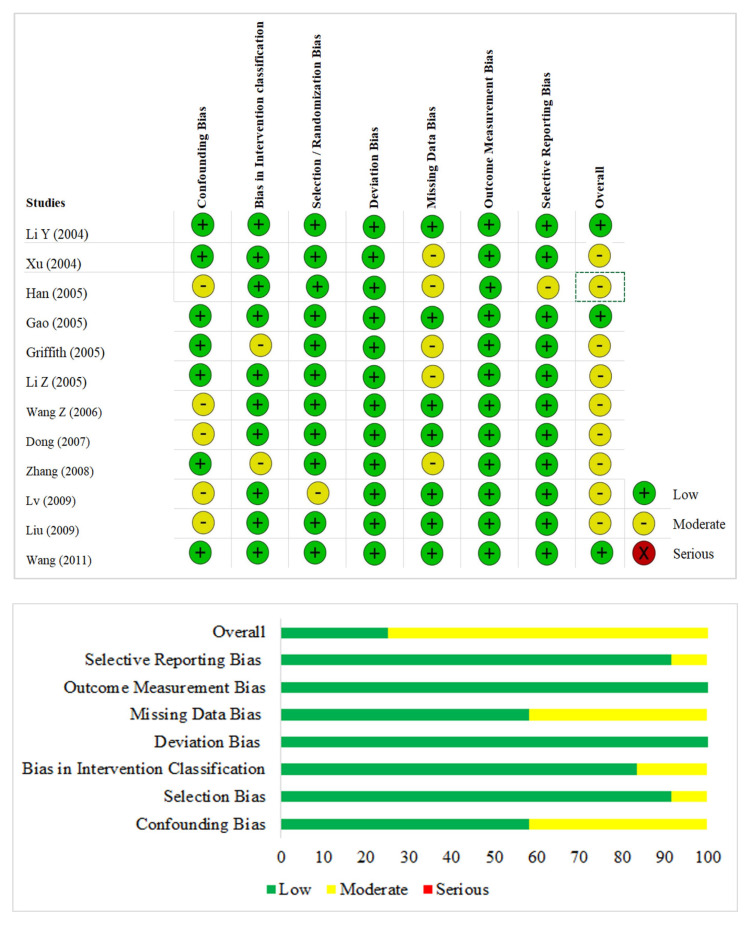
Risk of bias of the included studies based on ROBINS tool of Cochrane Collaboration.

The mean age of the 1728 patients in the included studies was 33.41 years (±4.93 years). The mean dosage of CS administered was 4.64 g (±4.7 g) which was administered for a mean duration of 29.91 days (±12.3 days). All the patients included in the analysis were from China and Hong Kong. The range of publication dates was from 2004–2011. Ten of the 12 included studies were cohorts while the remaining 2 studies [18,24] were nested case–control studies. The mean duration of follow-up among the included studies was 16.3 months. Methylprednisolone was the most commonly used CS treatment in the included studies. The prednisolone doses presented in two studies [19,25] were converted to methylprednisolone using a 0.8 conversion factor. The cumulative dose of various types of CS was converted to methylprednisolone-equivalent doses for analysis. We noted variability in the duration and dosage of CS exposure among the included studies. The risk of bias in the included studies is presented in Figure 2. None of the included studies demonstrated a high risk to warrant exclusion from the analysis.

**Figure 3:**
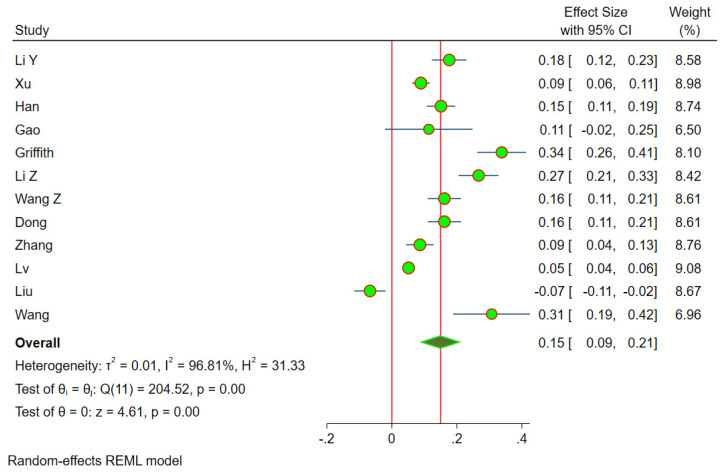
Dose–response meta-analysis forest plot of the individual studies included in the analysis demonstrating a significant risk of osteonecrosis of the femoral head with the incremental dosage of cumulative corticosteroids administered.

**Figure 4:**
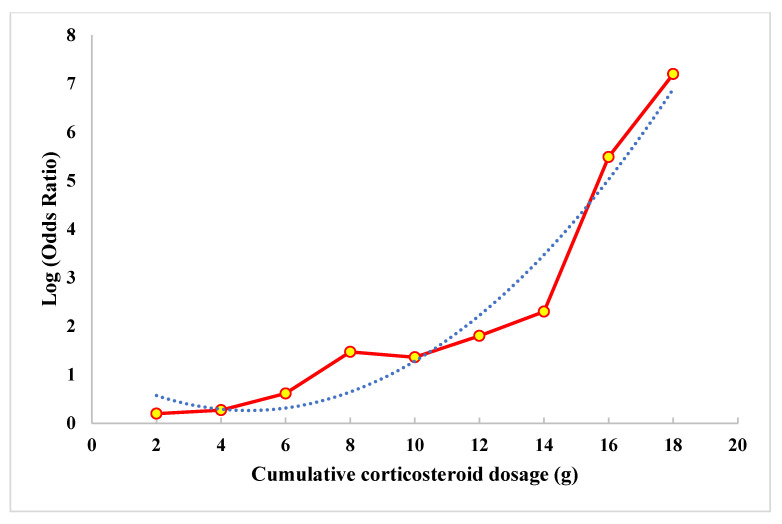
Relationship of risk of osteonecrosis of the femoral head with the dosage of corticosteroids estimated by restricted cubic spine model.

***Dose–response Meta-analysis:*** Twelve studies [18,19,20,21,22,23,24,25,26,27,28,29] were included in the dose–response meta-analysis. We noted significant heterogeneity among the included studies (i^2^ = 96.8%, *p* < 0.001), hence, we used the random effects model of dose–response meta-analysis as shown in Figure 3. The summary OR of osteonecrosis was 1.16 (95% CI 1.09–1.23, *p* < 0.001) per 2.0 g increase in cumulative CS usage. The relationship was non-linear polynomial as shown by the restricted cubic spline curve in Figure 4. It is evident from the spline curve that the risk of OFH rises once the cumulative dose of the CS used rises above 4 g.

**Figure 5:**
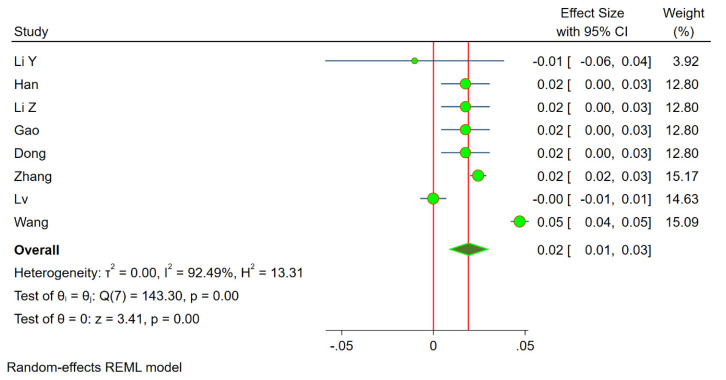
Duration–response meta-analysis forest plot of the individual studies included in the analysis demonstrating a significant risk of osteonecrosis of the femoral head with the incremental duration of cumulative corticosteroids administered.

**Figure 6:**
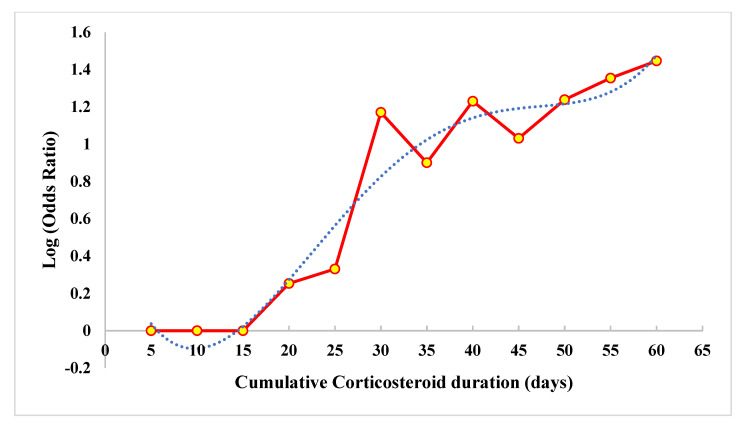
Relationship of risk of osteonecrosis of the femoral head with the duration of corticosteroids estimated by restricted cubic spine model.

***Duration–response Meta-analysis:*** Four studies [20,25,26,27] were excluded from the analysis since they did not provide the cumulative duration of CS usage. Eight studies [19,21,22,23,24,26,28,29] were included in the duration response meta-analysis. We noted significant heterogeneity among the included studies (i^2^ = 92.4%, *p* < 0.001), hence, we used the random effects model of dose–response meta-analysis as shown in Figure 5. The summary OR of osteonecrosis was 1.02 (95% CI 1.01–1.03, *p* < 0.001) per 5 days of increase in the cumulative duration of CS usage. The relationship was non-linear polynomial as shown by the restricted cubic spline curve in Figure 6. It is evident from the spline curve that the risk of OFH rises once the cumulative duration of the CS treatment is more than 15 days.

**Figure 7:**
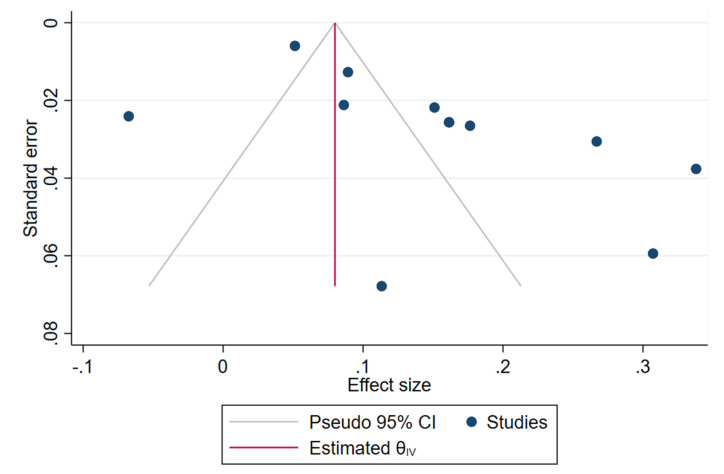
Publication bias in the included studies.

***Sensitivity Analysis and Funnel Plots:*** We analyzed the robustness of the results by excluding studies with the highest positive and negative impact on the overall pooled summary effects. However, the heterogeneity among the included studies was due to the wider confidence intervals of the included studies. We also analyzed the heterogeneity with a leave-one-out analysis to identify the impact of the individual studies on the overall effect size and the heterogeneity values obtained. We did not find any single study to alter the overall inference obtained from the pooled analysis. On further analysis of publication bias with funnel plot as shown in Figure 7, we noted evident publication bias noted by the asymmetry of the distribution of the included studies on either side of the 95% CI axis. Most of the contributions in the included studies were from China while the rest of the world did not analyze this use of CS with OFH since China was the epicenter of the pandemic outbreak with a high risk of complications due to the unavailability of standardized protocols during the pandemic scenario, while the rest of the world learned from the Chinese experience in order to manage the pandemic effectively. We made a trim-and-fill analysis to identify the number of studies needed to compensate for the missing studies. We identified that the addition of 3 more studies compensated for the publication bias but that did not alter the overall inference obtained from the current analysis.

## 4. Discussion

A dose–response meta-analysis of the effects of CS during the SARS outbreak was used as the basis for risk classification and screening strategies for osteonecrosis of the femoral head following CS therapy for COVID-19. This analysis was used as a basis for risk classification and screening strategies. In this study, we investigated how the effects of CS varied over the course of time. The findings of the analysis were utilized as the basis for the formulation of these new policies and procedures. The risk of developing osteonecrosis is determined by the meta-analysis, which takes into account two factors: the total amount of CS that were taken and the entire period of time that therapy was carried out. Patients who are at a higher risk of developing osteonecrosis, such as those who have been on high-dose CS for an extended period of time, may require closer monitoring and earlier management in order to avoid or delay the disease’s onset. Patients who have been on high-dose CS for an extended period of time may also be at a higher risk of developing osteonecrosis. People who are at a greater risk of acquiring osteonecrosis typically have a more severe case of the condition than patients who are at a lower risk of developing osteonecrosis. Clinical examinations, imaging investigations, and laboratory tests are all examples of screening procedures that can be utilized to discover early indicators of osteonecrosis. There is a possibility that one or more of these screening processes will be utilized. It is possible to enhance patient outcomes by preventing or delaying the progression of the illness through early detection and prompt action. This is one way in which early detection and prompt action can be used. Increasing the positive outcomes for patients is one way to achieve this goal. It is important to note that while this meta-analysis can provide guidance for risk stratification and screening, individual patient factors such as medical history, underlying health conditions, and other medications used may also have an influence on the risk of developing osteonecrosis.

Having not identified a specific drug to treat COVID-19, symptomatic support remained the most effective treatment in managing the manifestations of the disease. The use of CS in the management of acute respiratory distress syndrome (ARDS) due to severe COVID-19-induced pneumonia remains controversial [30]. Although it is a well-known fact that CS are useful in the management of ARDS since they have the potential to reduce inflammation and improve respiratory function, the meta-analysis by Stockman LJ et al. [31] has shown that the use of CS was harmful. In the preliminary data on the retrospective cohort study from China, CS use was most frequently found in the patients who died (48%) rather than those who survived (23%) [32]. The above results were thought to be due to the overestimation of the benefits of using CS in more critically ill patients with ARDS with inherently poor prognoses [33]. Hence, with a lot of confounders to the results obtained in various studies and the selection bias in the population involved, solid scientific evidence could not be obtained on the use of CS in COVID-19 which made the WHO and Centre for Disease Control and Prevention (CDC) recommend against the routine use of CS in the management of ARDS in COVID-19 pneumonia unless otherwise indicated for Critical COVID-19 patients requiring life-sustaining therapies such as mechanical ventilation or vasopressor therapy and Severe COVID-19 with oxygen saturation <90% on room air, respiratory rate >30 breaths per minute for adults, or with signs of severe respiratory distress [34,35,36].

Zha et al. noted that 35.4% of patients with COVID-19 received 2 g of CS within 5 days of its management [37]. Moreover, no correlation was noted between CS usage and viral clearance or hospital stay, or symptom resolution based on Cox proportional hazard regression analysis in the above study. In situations where the advantages are uncertain, the incidence of complications was definite. Hui et al. [19] noted that almost 40% of the patients who received CS therapy developed OFH. Some studies noted an incidence of OFH in cases who received CS for less than 4 weeks in the management of SARS also which made them propose that the SARS virus by itself could be an independent risk factor in the development of OFH through their S protein [38,39]. It is prudent that a strong systemic inflammatory response in patients with varying levels of hypoxia could also precipitate OFH. COVID-19 patients also suffer from similar pathological processes and hence we do not consider the usage of CS therapy in those critically ill patients to be irrational. However, one must be warned about the complications such as osteoporosis, hyperglycemia, cardiovascular disease, adrenal suppression, Cushing’s syndrome, and immunosuppression with the routine use of this therapy for COVID-19 [40]. In a comparative study, bacterial and fungal infection rates were significantly higher in the CS group compared to the non-steroid counterparts [41]. Glucocorticoid therapy-induced invasive fungal infections were also globally reported in COVID-19 [42,43]. Having noted all the controversies in glucocorticoid therapy, the major findings in our meta-analysis were as follows:

1. The risk of osteonecrosis increases when the cumulative dose of CS used is above 4 g at an OR of 1.16 (95% CI 1.09–1.23, *p* < 0.001) per 2.0 g increase in the cumulative dose of CS usage.
2. Similarly, the risk of osteonecrosis increases when the cumulative duration of CS therapy is above 15 days at an OR of 1.02 (95% CI 1.01–1.03, *p* < 0.001) per 5 days of increase in the cumulative duration of CS usage.

Apart from the cumulative dose of exposure, logistic regression analysis by Shen et al. [44] noted a correlation between the maximum daily dose of glucocorticoid therapy and the risk of OFH, thereby highlighting the importance of controlling the daily dosage. Similarly, an increase in the incidence of OFH by 3.6% was noted for every 10 mg increase in the dose of CS used [6]. Besides the cumulative dose of exposure, Zhao et al. [45] in their dose–response meta-analysis noted the risk ratio of 1.29 for the development of OFH for every 10 days of CS therapy and the relationship was non-linear, thereby recommending a reduction of the total duration of the therapy to prevent OFH. Our meta-analysis proves that an increase in the cumulative dose and the duration of the CS therapy increased the risk of developing OFH, hence, we recommend low-dose and short-term use of CS in critically ill patients of COVID-19, ideally, less than 4 g and less than 15 days, respectively. Besides glucocorticoid therapy, a COVID-19-induced prothrombotic state could also be a reason for the raised incidence of OFH noted, which further makes our analysis more crucial, thereby making the glucocorticoid therapy complicate it further and hence the risk that goes with it [46]. Although SARS-CoV-2 is 76% similar in genomic sequencing to SARS-CoV-1 [47], there exists variation in their pathogenesis that involves microvascular as well as large vessel thrombosis mediated through a unique thromboinflammatory cascade triggered by the infection that compounds the risk of events such as OFH despite the limited use of CS used in their therapeutic regimen [46]. Hence, the interpretation of our results must be taken with caution with respect to the unique differences between SARS and SARS-CoV-2, thereby making the risk of OFH even higher than SARS due to the predisposition to hypercoagulability in COVID-19 [48].

**Figure 8:**
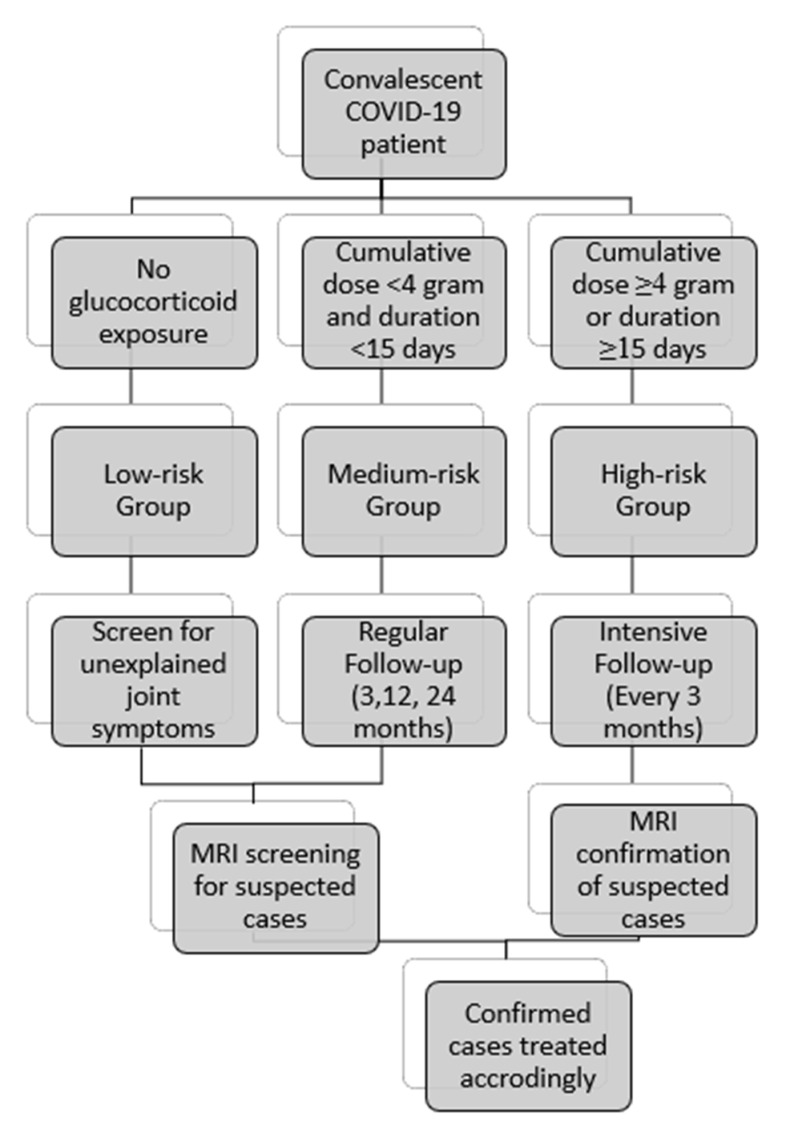
Screening protocol for OFH in convalescent COVID patients.

Apart from the dose and duration, there might be other patient-related factors that also play a role in enhancing the risk in the individual for OFH such as age, sex, comorbid illness, smoking status, alcohol intake, etc. Although there were reports of OFH in elderly individuals even with a single shot of CS [49], we also noted several reports of young individuals with OFH post-COVID-19 illness [50]. In a prospective study by Veizi et al. [51] among the 472 COVID-19 patients used to evaluate the incidence of OFH, they noted that individuals with a history of smoking and alcohol use have a significantly higher risk for the development of OFH. Similarly, the comorbid illness of the individual might also contribute to the risk of OFH in post-COVID-19 illness which needs further evaluation. For the patient who has undergone treatment in the past waves of COVID-19, a screening strategy has been devised for early identification of OFH and to address it appropriately. Since reports of OFH were made even in low-dose and short-term use of CS [52], we recommend screening all cases of COVID-19 who have undergone CS therapy as per the following protocol as shown in Figure 8. Apart from CS use in the management of COVID-19, patients with pre-existing diseases such as chronic respiratory or dermatological ailments that required long-term use of CS were also included in the medium-risk group for regular follow-up evaluations.

OFH has no optimal treatment method to date, thereby causing the patient to become disabled once the bone collapses. The choice of treatment is mainly based on the estimation of the risk of collapse of the femoral head [53]. Non-operative measures are ineffective to prevent cases with a high risk of collapse and they require hip-preserving procedures such as core decompression, osteotomies around the hip, and vascular or non-vascular grafts to give a conducive environment for the femoral head to recover from the ischemic damage [54]. For a patient with advanced collapse with osteoarthritis, total joint replacement becomes necessary [53].

Our study has certain limitations. Despite the similarity in the pathogenesis and available treatment methods of SARS and COVID-19, inherent variation lies in the viral genome and the prothrombotic cascade triggered by COVID-19 could alter the incidence of OFH despite the common exposure of CS in their management protocols. Concerning the availability of long-term data in SARS patients, we have extrapolated the SARS model to screen the development of OFH in convalescent COVID-19 individuals. CS usage has both cumulative and idiosyncratic effects. Though our analysis elaborated on the critical cut-off of cumulative dosage of CS to be used, we could not identify the critical cut-off of daily dosage of CS to reduce the risk of OFH due to the lack of data across the included studies. However, some of the prospective studies evaluating the incidence of OFH among post-COVID-19 individuals validated our screening strategy where they noted less risk of OFH upon individuals with lesser CS usage and lesser duration of hospitalization compared to individuals who developed OFH post-COID-19 at 2 years follow-up [51]. Apart from the dose and duration, there might be other patient-related factors that have contributed to the risk of OFH such as age, sex, comorbid illnesses, etc., that were not analyzed in the current study.

## 5. Conclusions

Patients with a cumulative dose of CS therapy of more than 4 g and a total duration of exposure of more than 15 days demonstrated a significant risk of development of OFH. Appropriate and frequent screening of these individuals at regular intervals would help in the identification of the disease at an early stage in order to treat them appropriately.

## Figures and Tables

**Table 1:** Characteristics of the studies included in the review (*n* = 12).

S. No.	Author	Year	Country	Study Design	Mean Age (Years)	M:F	Case/ Control	Follow-Up (Months)	Mean CS Usage (Grams)	Mean Duration of Usage (Days)
1	Li Y et al. [23]	2004	China	Cohort study	29	12:28	33/7	3	4.9	24
2	Xu et al. [27]	2004	China	Cohort study	31.5	61:32	30/63	2.5	7.2	30
3	Han et al. [21]	2005	China	Cohort study	32.6	43:37	21/59	6	4	36
4	Gao et al. [24]	2005	China	Nested case-control study	30	12:28	12/28	12	4.9	24
5	Griffith et al. [25]	2005	Hong Kong	Cohort study	33	99:155	12/242	6.7	3.2	-
6	Li Z et al. [21]	2005	China	Cohort study	33	131:420	539/12	12	5.8	39
7	Wang et al. [18]	2006	China	Nested case-control study	47	-	39/19	4	4	33
8	Dong et al. [22]	2007	China	Cohort study	37.2	41:51	6/84	12	1.5	11
9	Zhang et al. [29]	2008	China	Cohort study	32.1	34:80	43/71	6.5	4.1	29
10	Lv et al. [19]	2009	China	Cohort study	30	30:41	41/30	36	4.8	34
11	Liu et al. [20]	2009	China	Cohort study	30.6	-	34/69	6	4.4	28
12	Wang et al. [26]	2011	China	Cohort study	35	95:137	65/167	90	6.9	41

The cumulative doses of corticosteroids (CS) used across various studies were represented in methylprednisolone-equivalent doses.

## Data Availability

The data is contained within the manuscript.

## Supplementary Materials

The following supporting information can be downloaded at: https://www.mdpi.com/article/10.3390/life13040907/s1, Table S1: Search strategy used in PubMed database (Search date: 30 December 2022).
