# Supplementary material for: Dose–Response Meta-Analysis of Corticosteroid Effects in SARS Outbreak: A Model for Risk Stratification and Screening Strategy for Osteonecrosis of Femoral Head Post-Corticosteroid Therapy for COVID-19
Source: Life (Basel). 2023 Mar 29;13(4):907. doi: 10.3390/life13040907 (PMC10143798; doi:10.3390/life13040907)
Supplement: Supplementary file 1 [file life-13-00907-s001.zip › life-2189880-supplementary.pdf]

Supplementary Table S1:

Search strategy used in PubMed database (Search date; 30 December 2022)

Search Topic	**#**	**Search Terms**
Population: *Human subjects with SARS developing osteonecrosis of femoral head*	#1	("Severe Acute Respiratory Syndrome Virus"[Mesh]) OR ("SARS-CoV"[Mesh]) OR (SARS-Related Coronavirus[All Fields]) OR (SARS[All Fields])
	#2	("hip joint"[MeSH]) OR ("hip joint"[All Fields]) OR ("femoral head"[All Fields]) OR ("avascular necrosis"[MeSH]) OR ("osteonecrosis"[MeSH]) OR ("AVN"[All Fields])
	#3	#1 AND #2
Intervention: *Steroid therapy*	#4	("glucocorticoids"[Mesh]) OR ("corticosteroids"[Mesh]) OR ("steroids"[Mesh]) OR ("cortisol"[All Fields]) OR ("steroids"[All Fields]) OR ("corticosteroids"[All Fields]) OR ("glucocorticoids"[All Fields])
	#5	("hydrocortisone"[Mesh]) OR ("prednisolone"[Mesh]) OR ("methylprednisolone"[Mesh]) OR ("prednisolone"[All Fields]) OR ("methyl prednisolone"[All Fields]) OR ("hydrocortisone"[All Fields])
	#6	#4 AND #5
	#7	#3 AND #6
Study design: *Prospective or Retrospective Cohort Studies*	#8	("prospective studies"[MeSH Terms] OR "prospective studies"[All Fields] OR "retrospective studies"[MeSH Terms] OR "retrospective studies"[All Fields] OR "retrospective study"[All Fields])
	#9	#7 AND #8
	#10	("biography"[Publication Type] OR "comment"[Publication Type] OR "directory"[Publication Type] OR "editorial"[Publication Type] OR "festschrift"[Publication Type] OR "interview"[Publication Type] OR "lecture"[Publication Type] OR "legal case"[Publication Type] OR

	“legislation”[Publication Type] OR “letter”[Publication Type] OR “news”[Publication Type] OR “newspaper article”[Publication Type] OR “patient education handout”[Publication Type] OR “popular work”[Publication Type] OR “congress”[Publication Type] OR “consensus development conference”[Publication Type] OR “consensus development conference, nih”[Publication Type] OR “practice guideline”[Publication Type]) OR “Review”[Publication Type])
#11	#9 NOT #10
